# Predicting a local recurrence after breast-conserving therapy by gene expression profiling

**DOI:** 10.1186/bcr1614

**Published:** 2006-10-30

**Authors:** Dimitry SA Nuyten, Bas Kreike, Augustinus AM Hart, Jen-Tsan Ashley Chi, Julie B Sneddon, Lodewyk FA Wessels, Hans J Peterse, Harry Bartelink, Patrick O Brown, Howard Y Chang, Marc J van de Vijver

**Affiliations:** 1 Department of Radiation Oncology, The Netherlands Cancer Institute/Antoni van Leeuwenhoek Hospital, Plesmanlaan 121, 1066 CX Amsterdam, The Netherlands; 2 Department of Diagnostic Oncology, The Netherlands Cancer Institute/Antoni van Leeuwenhoek Hospital, Plesmanlaan 121, 1066 CX Amsterdam, The Netherlands; 3 Department of Experimental Therapy, The Netherlands Cancer Institute/Antoni van Leeuwenhoek Hospital, Plesmanlaan 121, 1066 CX Amsterdam, The Netherlands; 4 Department of Biochemistry, Stanford University School of Medicine, Stanford, CA 94305, USA; 5 Program in Epithelial Biology, Howard Hughes Medical Institute, Stanford University School of Medicine, Stanford, CA 94305, USA

## Abstract

**Introduction:**

To tailor local treatment in breast cancer patients there is a need for predicting ipsilateral recurrences after breast-conserving therapy. After adequate treatment (excision with free margins and radiotherapy), young age and incompletely excised extensive intraductal component are predictors for local recurrence, but many local recurrences can still not be predicted. Here we have used gene expression profiling by microarray analysis to identify gene expression profiles that can help to predict local recurrence in individual patients.

**Methods:**

By using previously established gene expression profiles with proven value in predicting metastasis-free and overall survival (wound-response signature, 70-gene prognosis profile and hypoxia-induced profile) and training towards an optimal prediction of local recurrences in a training series, we establish a classifier for local recurrence after breast-conserving therapy.

**Results:**

Validation of the different gene lists shows that the wound-response signature is able to separate patients with a high (29%) or low (5%) risk of a local recurrence at 10 years (sensitivity 87.5%, specificity 75%). In multivariable analysis the classifier is an independent predictor for local recurrence.

**Conclusion:**

Our findings indicate that gene expression profiling can identify subgroups of patients at increased risk of developing a local recurrence after breast-conserving therapy.

## Introduction

Breast-conserving therapy (BCT) is a well-established treatment modality for early (stages I and II) breast cancer. The treatment consists of complete surgical excision of the tumor followed by whole breast irradiation. In multiple randomized trials comparing BCT with mastectomy, their equality in overall survival has been shown [1-7]. A large population-based Danish series showed that in different age categories (less than 35 years, 35 to 39 years, 40 to 44 years and 45 to 49 years) of young patients, survival was not negatively influenced by BCT [8]. However, local recurrence rates were significantly higher after BCT than after mastectomy in all series. The standard treatment for a local recurrence is a salvage mastectomy, which negates the original cosmetic intentions of BCT. More importantly, a recent meta-analysis by the Early Breast Cancer Trialists' Collaborative Group showed a negative impact of a local recurrence on survival [9]. They concluded: 'Differences in local treatment that substantially affect local recurrence rates would, in the hypothetical absence of any other causes of death, avoid about one breast cancer death over the next 15 years for every four local recurrences avoided, and should reduce 15-year overall mortality.'

Identifying patients at high risk for local recurrence in advance and individualizing treatment in these patients (for example, a higher radiotherapy dose ('boost') or a primary mastectomy) is desirable. Several risk factors for local recurrence have been recognized [10-17]. Margin status, young age, an incompletely excised extensive intraductal component and inadequate radiotherapy dose (boost) have been identified as important risk factors for local recurrence [18]. Adjuvant systemic treatment (chemotherapy or hormonal therapy) is known to reduce the risk of a local recurrence [12,14,16].

Previous studies have shown the ability to predict distant-metastasis-free and overall survival in breast cancer with the use of microarray analysis [19-25]. Mechanisms of recurrence in the breast are not necessarily the same as mechanisms involved in distant metastasis. Theoretically, radioresistance of the tumor cells would be an important factor in local recurrence after BCT but not necessarily in distant metastasis.

Previous analyses of gene expressions patterns in a series of 295 early-stage breast cancer patients have identified gene expression signatures that powerfully predict the risk of distant metastasis and mortality [22,23,26,27]. In the present study we used a supervised approach to search for gene expression signatures that predict the risk of local recurrence after BCT in a series of 161 early-stage breast cancer patients.

## Materials and methods

### Tumor samples and patients

This analysis is based on previously reported gene expression profiles of tumors from a series of 295 stage I and II breast cancer patients treated at the Netherlands Cancer Institute between 1984 and 1995 [23]. All patients were under 53 years of age at the time of diagnosis. For this study all the patients from this series who received BCT (*n *= 161) were selected. BCT consisted of a wide local excision and axillary lymph node dissection followed by whole breast irradiation (median dose 50 Gy, range 50 to 54 Gy; the use of 6, 8 or 18 MV photons depending on breast diameter); 144 patients received a boost to a median dose of 15 Gy, 98 patients received a low boost (14 to 18 Gy), and 46 patients received a higher boost (20 to 26 Gy). The boost technique was delivered using ^192^I (iridium) implantation (88 patients), electrons (31 patients) or photons (25 patients). Pathological margins were assessed as free of tumor in 134 patients, focal involvement by invasive carcinoma only in 3 patients, focal involvement by ductal carcinoma *in situ *(DCIS) only in 5 patients, and focal involvement by both DCIS and invasive carcinoma in 4 patients; in 8 patients there was more than focal involvement of the margins by DCIS, or the degree of margin involvement could not be determined with certainty. For four patients there was invasive carcinoma at the margin, but the extent of involvement could not be determined with certainty, and for three patients this was the case for both invasive carcinoma and a DCIS component. Of all 27 patients with any extent of involved margins, 2 did not receive a boost, 6 received a low boost and 19 a high boost of radiotherapy. Follow-up assessment was at least yearly, including mammography. Seventeen patients developed a local recurrence; the resection margins of the primary tumor had been free of any tumor for 15 of these 17 patients. One patient who later developed a local recurrence had focal involvement of the resection margins with invasive carcinoma, and one patient had focal involvement with DCIS. The 10-year local-recurrence-free percentage was 85% (median follow-up for all patients was 7.7 years, and 8.5 years for alive patients).

On the basis of the microarray identification number, patients were alternately assigned to the training set (that is, developing a predictor of local recurrence) or to the validation set. This was done separately for locally recurring and locally tumor-free patients, thereby creating almost equal-sized sets with balancing for local recurrence.

Patient characteristics for both the training and validation series are equally divided and are shown in Table 1. In Table 2 characteristics are listed for patients with and without a local recurrence. A full clinical data sheet is available online as Additional file 1, on [28] and on the paper's home page [29].

### Microarray procedures

RNA isolation, labeling of complementary RNA, and hybridization to 25,000-element oliogonucleotide microarrays, and measurement of expression ratios were performed as described previously [23].

The microarray data for analysis of the 70-gene prognosis profile were obtained with the use of the same 25,000-element oligonucleotide array; the correlation coefficient of the expression of these 70 genes with the good prognosis profile was calculated as reported previously [22]. The two other gene lists used are derived from Stanford cDNA arrays [30]. The correspondence between genes represented by Stanford cDNA microarrays and Rosetta/NKI oligonucleotide microarrays were established by using Unigene identifiers (build 158, release date 18 January 2003 for the wound signature, as this was the original mapping carried out for the validation of the wound signature [26]). The primary hypoxia analysis was performed at a later time. We used build 172, release date 17 July 2004 [27]. Because of the relatively small number of genes, probes on the Rosetta/NKI array that were mapped to the same unigene cluster were averaged.

The entire expression database for all 295 patients is publicly available online [28], as are the files containing the expression ratios for the specific experiments (70 genes (Additional file 2), hypoxia signature (Additional file 3) and wound signature (Additional file 4) [29].

### Class prediction and gene selection *de novo*

The first method we used for predicting local recurrence is PAM (prediction analysis for microarrays): class prediction as described by Tibshirani and colleagues [31]. This method shifts the mean expression level of each gene (possibly after transformation) for each class (for example local recurrence) towards the overall mean expression level for all classes by a fixed standardized difference (shrunken centroids). The class for which the shrunken centroid most closely reaches the observed expression pattern of a certain patient by using a Pearson correlation is then the predicted class for that patient. For a given shrunken centroid only those genes for which the shrunken means still differ from the overall mean will contribute to the distance between centroids and any individual tumor sample's expression pattern. The standardized difference, and thereby the number of relevant genes, is chosen by minimizing the prediction error using 10-fold balanced, leave-10%-out cross-validation within the training set. The same method is then used to predict classes for new samples (validation set).

### Gene set enrichment analysis

Subramanian and colleagues [32] have described a method that analyzes whether or not predefined gene sets (representing biological pathways) are coordinately and significantly upregulated or downregulated between samples that represent different biological or clinical entities [32]. The program corrects for multiple testing. We applied this method to our data set and analyzed gene set C2 ('functional gene set'). The software and gene set are available for download [33]. C2 represents 504 gene sets. Standard parameters were used for the analysis. The threshold for false discovery rate is 25% before a gene set is called significantly upregulated or downregulated.

### Established gene expression profiles and methods of supervised analysis

The following previously established gene expression profiles were used to find a classifier for local recurrence after BCT:

1. The wound-response gene expression signature is a characteristic pattern of expression of a set of genes that have been identified as being induced or repressed in response to bovine serum, a surrogate wound environment [30,34]. This so-called 'core serum response' has shown to be a strong predictive factor for outcome in cancer (breast, head and neck, lung and gastric) using metastasis-free and overall survival as endpoints [26,30]. It is important to realize that a particular pattern of expression of these genes, not just the genes themselves, predicts outcome.

2. The 70-gene prognosis profile was originally trained to predict metastasis within 5 years ('poor prognosis' group), or no metastasis within 5 years ('good prognosis' group) [22].

3. The hypoxia profile is a gene expression classifier from an *in vitro *experiment in which epithelial cells are exposed to hypoxia [27].

First, these gene expression profiles were used in their original form and tested for their value in predicting local recurrence after BCT in the complete set of 161 patients.

Second, we optimized these profiles by using the clinical data on local recurrence.

Using the training data set, we calculated for each of the three profiles the average pattern of expression of the genes that comprised each profile, for patients who developed a local recurrence (local recurrence profile or local recurrence centroid). For all patients in the training and validation set we then calculated the non-centralized Pearson correlation to each of the three average patterns of expression (local recurrence profile or local recurrence centroid). A correlation value between -1 and +1 is thereby derived for all patients and indicates the similarity in gene expression between an individual patient (tumor) and the average gene expression of tumors from patients in the training set who developed a local recurrence.

**Figure 1:**
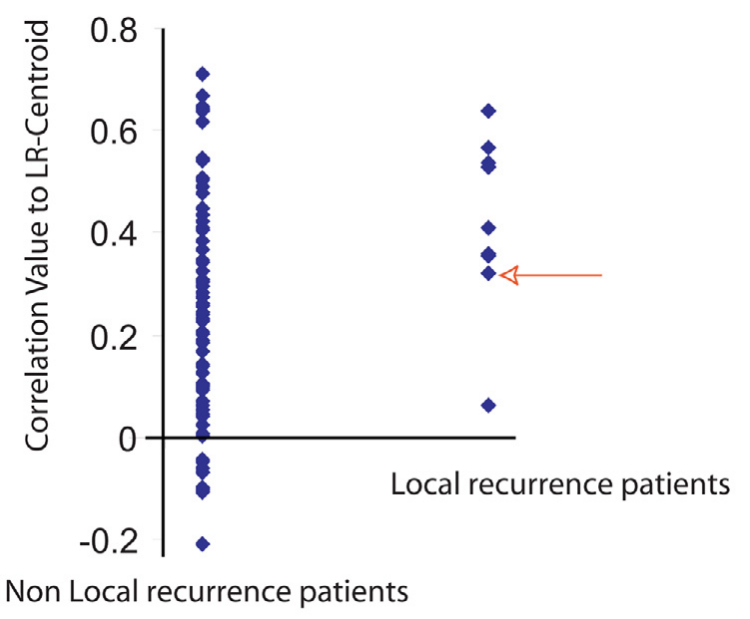
Local recurrence Wound-signature correlation plot for the training set. Red arrow indicates optimal threshold.

**Figure 2:**
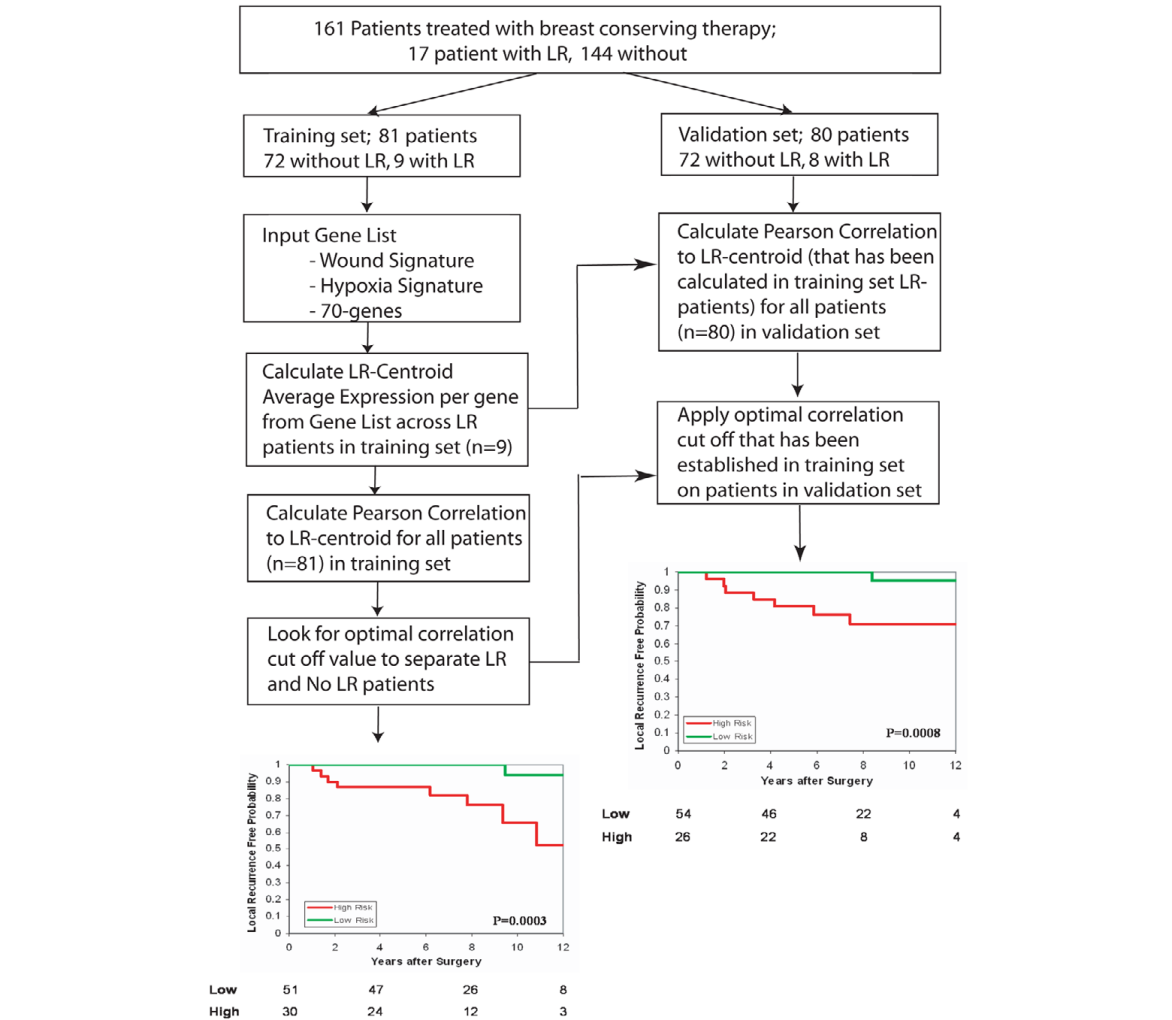
Scheme for training and validation, including Kaplan–Meier curves for local-recurrence-free survival.

For each of the three signatures a plot of these correlation values (see Figure 1 for the core serum response genes) is drawn for the training set and shows the difference in expression between patients who developed a local recurrence and those who did not. On the basis of the difference in the distribution of these correlation values, we selected the maximal threshold, misclassifying only one out of nine local recurrences in the training set. Patients with a correlation value above the threshold were classified as high-risk, and patients below the threshold as low-risk. Except for the gene selection process, this is the same procedure as followed by van't Veer and colleagues [21]. The local recurrence profile and its associated threshold value were subsequently validated on the validation set. The methods for training and validation are depicted schematically in Figure 2.

### Statistics

All patients who developed a local recurrence were considered; 16 patients developed a local recurrence as a first event and in 1 patient local recurrence occurred concurrently with distant metastases. Local-recurrence-free percentages were calculated with the Kaplan–Meier method (log-rank test for comparison). All Kaplan–Meier curves are available as supplementary data in Additional file 5. Comparison of categorical variables between groups was performed with a χ^2 ^test. For the multivariable analysis we used a Cox regression model. All *p *values are two-sided. All correlations were calculated by the non-centralized Pearson method. For analysis we used Winstat^® ^for Excel (R. Fitch Software, Staufen, Germany), SPSS 13.0^® ^(SPSS Inc., Chicago, IL, USA) and Microsoft Excel^® ^(Microsoft Corporation) were used. The PAM analysis was performed with version 1.2 (November 2003) [31,35].

Additional material and methods are available described in Additional file 9.

## Results

In a previously reported series of 295 patients with stage I and II breast cancer diagnosed at age less than 53 years, 161 had been treated with BCT (the remaining 134 patients underwent modified radical mastectomy). Of these 161 patients, 17 developed a local recurrence. From the series of 161 patients, a training and validation set were selected as described in the Materials and methods section, distributing local recurrences equally. This resulted in a balanced distribution of year of treatment and major risk factors for local recurrence between the two groups as well (Table 1).

### Training and validation series

Nine out of 81 patients in the training set had developed a local recurrence at a median interval of 6.2 years (range 1.0 to 10.8 years); the median follow-up time for the patients in the entire training set was 7.9 years. Six out of nine local recurrence patients had received a boost (five patients received 15 Gy, one patient 25 Gy).

In the validation set, 8 out of 80 patients had developed a local recurrence at a median interval of 3.72 years (range 1.2 to 8.4 years); the median follow-up time for all patients in the validation set was 7.6 years. Seven out of these eight patients had received a boost (three patients received 15 Gy, four patients 25 Gy).

### Can a local recurrence classifier be identified by supervised classification using all genes?

We used the PAM algorithm with the training data set to search for gene expression profiles predictive of local recurrence, starting with a gene list that consisted of the top 5,000 most variably expressed genes with technically well-measured expression, represented by microarrays (out of 25,000). The performance of this approach, as evaluated by cross-validation, seemed to be poor: only 4 out of 9 local recurrences and 50 of the 72 non-local recurrences could be predicted accurately in the training set at this threshold (using a profile based on 684 genes). The use of fewer genes resulted in more accurate prediction for non-local-recurrence patients, but less accuracy for local-recurrence patients. In the validation series only 1 out of 8 local recurrences and 65 out of 72 non-local recurrences were predicted correctly (PAM analysis is provided in Additional files 6, 7, 8).

### Using predefined gene sets based on biological processes: gene set enrichment analysis

We used the GSEA (gene set enrichment analysis) software to analyze whether or not gene sets defined on the basis of biological processes or pathways were significantly correlated with local recurrence. We first ran the analysis on the full probe set (more than 23,000). We than ran the analysis on the same selection of genes used for the PAM analysis (the top 5,000). Neither the full probe set nor the top 5,000 probe set revealed any gene set to be significantly upregulated or downregulated in samples from tumors that recurred locally, after correction for multiple testing.

### Using previously identified gene expression signatures to predict local recurrence

We have previously shown that three distinct gene expression signatures can each be used to subdivide breast carcinomas into two groups that are associated, respectively, with good and poor distant-metastasis-free and overall survival: a '70-gene' prognosis profile [22,23], a wound-response signature [26,30] and a hypoxia-response signature [27]. We tested whether these three signatures were also associated with a difference in the risk of local recurrence. The 70-gene prognosis profile was originally trained to predict metastasis within 5 years ('poor prognosis' group) or no metastasis within 5 years ('good prognosis' group); in the current series of 161 patients, 97 patients had tumors with a poor prognosis profile and 64 patients had tumors with a good prognosis profile.

The wound-response signature was derived from an *in vitro *model for wound healing and can be used to classify tumors as having an 'activated wound-response signature' or a 'quiescent wound-response signature' [26,30]. In this series of tumors, 380 (out of 459) genes from the wound-response signature were identified; 65 tumors had an activated wound-response signature and 96 tumors had a quiescent wound-response signature.

The hypoxia-response profile is a gene expression classifier derived from an *ex vivo *model in which epithelial cells are exposed to hypoxia [27]. In this series of tumors, 123 (out of 168) genes from the hypoxia-response signature were identified. On the basis of the expression of these hypoxia-associated genes, the breast carcinomas in this study could be assigned to 36 cases with a hypoxia-response signature and 125 with a non-hypoxia signature.

Of these three signatures, only the wound-response signature showed some evidence (*p *= 0.04; log-rank) of predicting local recurrence (13% versus 19% at 10 years for quiescent versus activated).

### Building a classifier for local recurrence based on existing gene selections

Because the supervised approach using all genes (class prediction; PAM) failed and the previously identified gene expression signatures could not convincingly predict the risk of a local recurrence, we used genes from the original profiles and constructed a new classifier based on the relationship of their expression patterns to clinical outcome observed in our study (local recurrence or no local recurrence).

Each of these three signatures was originally defined for purposes other than the prediction of local recurrence, but we hypothesized that the optimization of each signature's pattern threshold by training on local recurrence data might improve their performance for this new purpose.

### Seventy-gene prognosis profile

Applying the method described in the Materials and methods section, a correlation value of 0.2364 was calculated as a cutoff point for the 70-gene expression profile as a predictor of local recurrence, resulting in one misclassified patient with local recurrence. The recurrence risks at 10 years were 7% and 28% for the low-risk and high-risk groups, respectively.

In the validation set the separation could not be reproduced (*p *= 0.34; log-rank). The sensitivity was 63% (5 out of 8) and the specificity 50% (36 out of 72). At 10 years the recurrence risk was 13% versus 14% for the low-risk group versus the high-risk group. Furthermore, more than half the patients were assigned by this classifier to the high-risk group (41/80).

### Hypoxia-response signature

For the hypoxia-response gene expression signature, the threshold that misses one patient with local recurrence seemed to be 0.254. In the training set the estimated 10-year recurrence risks for the low-risk and high-risk groups as determined by using this threshold were 10% and 19%, respectively. The performance of this signature on the validation set was not significant (*p *= 0.4). By employing this threshold on the validation set, 46 patients (58%) were classified as 'high risk' and 34 (42%) as low risk. At 10 years the recurrence risk was 13% versus 15% for low-risk versus high-risk, respectively. The sensitivity and specificity were 75% (6 out of 8) and 44% (32 out of 72), respectively.

### Wound-response signature: core serum response genes

The wound-response signature was first identified as a pattern of changes in gene expression induced by exposing fibroblasts to serum. A threshold value of 0.3179 for the correlation with the wound-response signature (as derived from the 380 available genes) resulted in one missed local recurrence in the training set. With the use of the threshold value for classification, 30 patients (37%) were classified as high-risk, and 51 (63%) as low-risk. In the training set, the 10-year recurrence risk was 6% for the 'low-risk' group and 35% for the 'high-risk' group. With this threshold, in the validation set 26 patients (33%) were classified as high-risk, and 54 (67%) as low-risk. The 10-year recurrence risk in the validation set was 5% for the low-risk group, in contrast with 29% for the high-risk group (*p *= 0.0008; log-rank). The predictive performance of the wound-response local recurrence signature on the validation set, using the threshold derived from the training set, is shown in Figure 2. The sensitivity of this classifier for predicting local recurrence in the validation set was 88% (7 out of 8) with a specificity of 74% (53 out of 72). The hazard ratio was 15 (95% confidence interval (CI) 1.8 to 123; log-rank 0.01). An overview of the sensitivity and specificity for the three supervised approaches is shown in Table 3.

### Cox regression: multivariable analysis

To assess the independent prognostic value of the supervised wound-response signature, it was tested in a Cox regression model that also included known clinico-pathological risk factors for local recurrence, using the independent validation data set. The factors that independently predicted a local recurrence in multivariable analysis in the EORTC 'boost versus no boost' trial were tested together with the local recurrence classifiers [17]. In the presence of age, diameter and boost treatment, all the gene expression classifiers were tested. Only the wound-response local recurrence signature (average local recurrence centroid) added significant prognostic information to the classical risk factors in predicting a local recurrence (hazard ratio 16 (95% CI 1.9 to 125); *p *= 0.01). Further results of the multivariable analysis are shown in Table 4.

## Discussion

We have shown that gene expression profiling using DNA-microarray analysis can be used in risk stratification for local recurrence after BCT in breast cancer patients. We tested several different methods: standard supervised analysis (PAM and GSEA, with local recurrence as the end point), profiles that have previously been used to predict distant metastasis and survival, and a combination of both approaches by supervising these previously established predictive profiles with clinical data on local recurrence.

The supervised wound-response signature derived by this approach is the only profile that could predict a local recurrence after BCT. This new method begins with a biologically defined gene set and constructs a classifier by a supervised learning procedure from the expression pattern of the selected gene set, using clinical data. This thereby uses not only the genes themselves but also the expression levels for the different genes in the signature that are correlated with the clinical end point (local recurrence). The power of this signature in predicting local recurrence was highly significant, even if we conservatively take into account the fact that we tried a total of seven methods, by applying a Bonferroni correction to the *p *value of 0.0008 associated with the supervised wound signature. It is important to note that the gene set derived from an *in vitro *model, the wound response, but not the wound-response gene expression signature itself, was adapted to derive a gene expression pattern predictive of a local recurrence in patients who were treated with BCT.

Gene expression profiling has been successfully applied to distinguish molecular subtypes in breast cancer and to predict metastases and overall survival [19-23,25]. More recently, gene expression profiling has also been used in trying to identify signatures predicting response to neo-adjuvant chemotherapy treatment [36,37], and prognosis after treatment with tamoxifen [38-41]. Because many patients are suitable candidates for breast conservation on the basis of low baseline risk for developing a local recurrence, this treatment has become standard for more than 60% of breast cancer patients. A recently published pooled analysis shows that local recurrence is associated with an increased risk of developing distant metastases and subsequent death from breast cancer [42]. These findings are similar to the recently published EBCTCG (the Early Breast Cancer Trialists' Collaborative Group) meta-analysis [9]. After BCT without radiotherapy, an absolute increase of 5.4% in breast cancer mortality was seen. Moreover, local recurrence requires treatment (usually salvage mastectomy) and is associated with anxiety for affected patients. It is therefore important to tailor BCT in such a way that the risk of local recurrence is kept as low as possible, while optimizing the quality of life, including the cosmetic appearance of the breast. To select patients who are likely to benefit from BCT, clinico-pathological parameters are currently used to stratify patients by risk of local recurrence. These existing prognostic factors are far from perfect, and additional or better predictors of local recurrence would be of great potential benefit. Aggressive treatment including a larger excision volume and/or a higher local radiotherapy dose ('boost') significantly decreases the risk at of local recurrence but results in impaired cosmetic outcome [43]. In patients considered to be at low risk of local recurrence, the boost dose could be forgone and in patients with a high risk a boost should be administered or even escalated. Furthermore, for patients at very high risk of local recurrence a primary mastectomy can be offered.

Because the number of patients and the number of local recurrence events was low in this study, these results must be interpreted with caution. Although the hazard ratio for a local recurrence in the validation series is high (15), the confidence interval is wide (by univariate analysis, 95% CI 1.8 to 123). Given the potential contribution of this prognostic signature to improve decision making in planning the treatment of breast cancer and an improved understanding of the pathogenesis of local recurrence, an independent test of its predictive value is a high priority. Recently, in The Netherlands, a randomized clinical trial started, to compare the standard radiotherapy dose (50 Gy whole breast and 16 Gy boost to the tumor bed) with an additional boost dose (26 Gy). In this trial, fresh frozen tumor samples will be collected. This trial will allow prospective validation of the signature.

## Conclusion

We have presented a method of integrating biologically derived gene expression profiles and clinical data to build a classifier for local recurrence after breast-conserving therapy. Although numbers of events are small and the resulting confidence intervals are wide, it is the first classifier based on microarray analysis to specifically predict a local recurrence after breast-conserving therapy. In our series the classifier is the most powerful predictor and is independent of clinical and pathological variables.

## Abbreviations

BCT = breast-conserving therapy; DCIS = ductal carcinoma *in situ*; GSEA = gene set enrichment analysis; PAM = prediction analysis for microarrays.

## Competing interests

The authors declare that they have no competing interests.

## Authors' contributions

DSAN conceived the method of building the gene expression profile specifically for local recurrence and performed the primary analysis. DSAN, BK, AAMH, JTAC, JBC, LFW, HJP, HB, POB, HYC and MJvdV analyzed and interpreted data. DSAN, AAMH and MJvdV wrote the paper. All authors read and approved the final manuscript.

## Supplementary Material

Additional file 1 An Excel file containing all clinical data, including all labels for various classifiers (both the original previously published labels as well as the local recurrence labels), and the correlation values (to the local recurrence centroids) for all patients. Click here for file

Additional file 2 An Excel file containing the expression data for the 70 genes for all patients (training (local recurrence and non-local recurrence) and validation set separately) and the calculated 70 genes local recurrence centroid data and correlation values for all patients to this centroid. Click here for file

Additional file 3 An Excel file containing the expression data for the hypoxia genes for all patients (training (local recurrence and non-local recurrence) and validation set separately) and the calculated hypoxia local recurrence centroid data and correlation values for all patients to this centroid. Click here for file

Additional file 4 An Excel file containing the expression data for the wound signature genes for all patients (training (local recurrence and non local recurrence) and validation set separately) and the calculated wound signature local recurrence centroid data and correlation values for all patients to this centroid. Click here for file

Additional file 5 An Excel file containing all Kaplan–Meier curves for the original classification and the supervised classifications. Click here for file

Additional file 6 A text file containing the expression data for the 5,000 most variable expressed genes on the array for all patients in the training set. Click here for file

Additional file 7 A text file containing the expression data for the 5,000 most variable expressed genes on the array for all patients in the validation set. Click here for file

Additional file 8 An Excel file containing the results from the PAM analysis. Click here for file

Additional file 9 A Word file containing supplementary additional details of material and methods. Click here for file

## Figures and Tables

**Table 1:** Characteristics of patients in the training and validation series

Characteristic	Training	Validation	All
No. of patients	81	80	161
Non-local recurrence	72 (89%)	72 (90%)	144 (89%)
Local recurrence	9 (11%)	8 (10%)	17 (11%)
Tumor size T1/T2	50/31 (62%/38%)	48/32 (60%/40%)	98/63 (61%/39%)
pN0/pN+	46/35 (57%/43%)	44/36 (55%/45%)	90/71 (56%/44%)
Grade I/II/III	22/26/33 (27%/32%/41%)	21/28/31 (26%/35%/39%)	43/54/64 (27%/33%/40%)
Age <40 years	62 (77%)	64 (80%)	126
Follow-up (years)	7.87	7.56	7.69
Metastasis	26 (32%)	23 (29%)	49 (30%)
Chemotherapy	28 (35%)	30 (38%)	58 (36%)
Hormonal therapy	10 (12%)	8 (10%)	18 (11%)
ER^+^/ER^-^	66/15 (81%/19%)	55/25 (69%/31%)	121/40 (75%/25%)
Median RT dose (Gy)	50	50	50
Median boost dose (Gy)	15	15	15
Boost, yes/no	70/11 (86%/16%)	74/6 (93%/7%)	144/17 (89%/11%)
Margins infiltrating carcinoma^a^	73, 4, 4 (90%, 5%, 5%)	72, 3, 5 (90%, 4%, 6%)	145, 7, 9 (90%, 4%, 6%)
Margins DCIS^b^	25, 48, 3, 5 (31%, 59%, 4%, 6%)	27, 41, 7, 5 (34%, 51%, 9%, 6%)	52, 89, 10, 10 (33%, 55%, 6%, 6%)

pN0, pathologically node negative; pN+, pathologically node positive; ER, estrogen receptor; RT, radiotherapy; DCIS, ductal carcinoma *in situ*.

^a^Results are given in the following sequence: radical, focally positive resection margin, extensively positive resection margin or positive resection margin not otherwise specified; ^b^results are given in the following sequence: no DCIS, radical, focally positive resection margin, extensively positive resection margin or positive resection margin not otherwise specified.

**Table 2:** Clinical characteristics by local recurrence versus non local recurrence

Characteristic	Non-local recurrence	Local recurrence	*p*^a^	All
No. of patients	144	17		161
Tumor size T1/T2	86/58 (67%/33%)	12/5 (71%/29%)	0.46	98/63 (61%/39%)
pN0/pN+	83/61 (58%/42%)	7/10 (41%/59%)	0.19	90/71 (56%/44%)
Grade I/II/III	41/47/56 (28%/33%/39%)	2/7/8 (12%/41%/47%)	0.16	43/54/64 (27/33/40%)
Age <40 years	28 (19%)	7 (41%)	0.074	35
Median follow-up (years)	7.68	9.31	0.014	7.69
Metastasis	40 (28%)	9 (53%)	0.004	49 (30%)
Chemotherapy	50 (35%)	8 (47%)	0.291	58 (36%)
Hormonal therapy	16 (11%)	2 (12%)	0.86	18 (11%)
ER^+^/ER^-^	109/35 (76%/24%)	12/5 (71%/29%)	0.42	121/40 (75%/25%)
Median RT dose (Gy)	50	50	0.47	50
Median boost dose (Gy)	15	15	0.22	15
Boost, yes/no	131/13 (91%/9%)	13/4 (76%/24%)	0.075	144/17 (89%/11%)
Margins infiltrating carcinoma^b^	129, 6, 9 (90%, 4%, 6%)	16, 1, 0 (94%, 6%, 0%)	0.365	145, 7, 9 (90%, 4%, 6%)
Margins DCIS^c^	49, 76, 9, 10 (34%, 53%, 6%, 7%)	3, 13, 1, 0 (18%, 76%, 6%, 0%)	0.65	52, 89, 10, 10 (33%, 55%, 6%, 6%)

pN0, pathologically node negative; pN+, pathologically node positive; ER, estrogen receptor; RT, radiotherapy; DCIS, ductal carcinoma *in situ*.

^a^Cox univariate; ^b^results are given in the following sequence: radical, focally positive resection margin, extensively positive resection margin or positive resection margin not otherwise specified; ^c^results are given in the following sequence: no DCIS, radical, focally positive resection margin, extensively positive resection margin or positive resection margin not otherwise specified.

**Table 3:** Performance of three different supervised analyses on validation series

Analysis	WS supervised	70-genes supervised	Hypoxia supervised
Sensitivity (percentage)	88	63	75
Specificity (percentage)	74	50	44
Low risk/high risk^a ^(percentage)	67/33	49/51	42/58
10-year LC low/high (percentage)	95/71	87/86	87/85
*p*^b^	0.0008	0.34	0.4

WS, wound signature; LC, local control.

^a^In validation set (percentages; *n *= 80); ^b^*p *value in validation set (log-rank).

**Table 4:** Cox regression analysis on local recurrence: clinical risk factors and wound signature (validation series only)

Risk factor	Significance	Hazard ratio for local recurrence	95% CI for hazard ratio	
			
			Lower	Upper
Age <40 years	0.39	2.542	0.30	21.7
Tumor size T2/T1	0.57	1.683	0.31	9.1
Boost treatment no/yes	0.83	1.343	0.09	20.2
Wound signature high risk/low risk	0.01	16	1.9	125

CI: confidence interval.
