# Supplementary material for: Predicting a local recurrence after breast-conserving therapy by gene expression profiling
Source: Breast Cancer Res. 2006 Oct 30;8(5):R62. doi: 10.1186/bcr1614 (PMC1779489; doi:10.1186/bcr1614)
Supplement: Additional file 9 — A Word file containing supplementary additional details of material and methods. [file bcr1614-S9.doc]

**Supplementary information**

**Material and Methods**

Mapping across different platforms

Two different platforms for micro-array analysis were used. The first platform is the Rosetta oligonucleotide 25k array. This array was used to perform the initial experiments. The 70-gene prognosis profile was also based on experiments that have been done on this array. In total 39 out of 78 patients used to define this profile, are also present in the current series, 20 in the training and 19 in the validation set.

The other two gene lists were derived from experiments done on the Stanford cDNA array. For our previous work [30] the Rosetta array was mapped to unigene cluster ID (build 158, release date Jan.18, 2003). For comparison, we kept on using this older version for both this paper and the validation of the Wound Signature [26].

Gene lists

For the PAM analysis we used the 5,000 most significantly expressed elements on the array. This is defined as follows. Elements are ranked by the number of patients that have a p-value of <0.01. The P-value for an element is defined as the significance level needed to state that a gene’s ratio is significantly different from 1, i.e. a difference between tumor sample and reference. This is based on a measurement error model derived from self-self experiments. Please see Roberts et al., *Science* **287**, 873-880 (2000) for further description of the error model.

Chang et al describe the core serum response genes (CSRG: [30]). This gene list consists of 459 genes. 442 probes on the Rosetta array representing 380 (out of 459) genes could be matched to the Stanford array.

The 70-gene prognosis profile is described by Van ‘t Veer et al. It is a supervised approach to predict distant metastasis within 5 years. As this list has been derived from the same platform, all 70 genes could be matched [22].

The hypoxia response is a profile that represents a hypoxia response *in vivo*. The gene list consists of 253 unique image clones on the cDNA Stanford array. Thirty-five of these could not be mapped to a unigene cluster. (Matching across unigene cluster was done using build 172, release date July 17^th^ 2004) [27].

The 218 remaining clones were mapped to 168 unique unigene clusters. The 168 unigene clusters represented 180 unique sequences on the Rosetta/NKI oligo array. Cross checking on gene names revealed 22 probes that could not confidently be contributed to genes in the original hypoxia signature. These were removed, resulting in 158 matched probes. These 158 were the matching probes to 123 unique unigene clusters. In order to overcome possible overestimation of unigene clones that were matched to more than one probe on the NKI array, the probes that were not uniquely matched to one unigene cluster were averaged.
